# Comparative BAC end sequence analysis of tomato and potato reveals overrepresentation of specific gene families in potato

**DOI:** 10.1186/1471-2229-8-34

**Published:** 2008-04-11

**Authors:** Erwin Datema, Lukas A Mueller, Robert Buels, James J Giovannoni, Richard GF Visser, Willem J Stiekema, Roeland CHJ van Ham

**Affiliations:** 1 Applied Bioinformatics, Plant Research International, PO Box 16, 6700 AA, Wageningen, The Netherlands; 2 Laboratory of Bioinformatics, Wageningen University, Transitorium, Dreijenlaan 3, 6703 HA Wageningen, The Netherlands; 3 Department of Plant Breeding and Genetics, Cornell University, Ithaca, New York 14853, USA; 4 United States Department of Agriculture and Boyce Thompson Institute for Plant, Research, Cornell University, Ithaca, New York 14853, USA; 5 Laboratory of Plant Breeding, Wageningen University, P.O. Box 386, 6700 AJ Wageningen, The Netherlands; 6 Centre for BioSystems Genomics (CBSG), PO Box 98, 6700 AB Wageningen, The Netherlands

## Abstract

**Background:**

Tomato (*Solanum lycopersicon*) and potato (*S. tuberosum*) are two economically important crop species, the genomes of which are currently being sequenced. This study presents a first genome-wide analysis of these two species, based on two large collections of BAC end sequences representing approximately 19% of the tomato genome and 10% of the potato genome.

**Results:**

The tomato genome has a higher repeat content than the potato genome, primarily due to a higher number of retrotransposon insertions in the tomato genome. On the other hand, simple sequence repeats are more abundant in potato than in tomato. The two genomes also differ in the frequency distribution of SSR motifs. Based on EST and protein alignments, potato appears to contain up to 6,400 more putative coding regions than tomato. Major gene families such as cytochrome P450 mono-oxygenases and serine-threonine protein kinases are significantly overrepresented in potato, compared to tomato. Moreover, the P450 superfamily appears to have expanded spectacularly in both species compared to *Arabidopsis thaliana*, suggesting an expanded network of secondary metabolic pathways in the *Solanaceae*. Both tomato and potato appear to have a low level of microsynteny with *A. thaliana*. A higher degree of synteny was observed with *Populus trichocarpa*, specifically in the region between 15.2 and 19.4 Mb on *P. trichocarpa *chromosome 10.

**Conclusion:**

The findings in this paper present a first glimpse into the evolution of Solanaceous genomes, both within the family and relative to other plant species. When the complete genome sequences of these species become available, whole-genome comparisons and protein- or repeat-family specific studies may shed more light on the observations made here.

## Background

The *Solanaceae*, or Nightshade family, is a dicot plant family that includes many economically important genera that are used in agriculture, horticulture, and other industries. Family members include the tuber bearing potato (*Solanum tuberosum*); a large number of fruit-bearing vegetables, such as peppers (*Capsicum spp*), tomatoes (*S. lycopersicum*), and eggplant (*S. melongena*); leafy tobacco (*Nicotiana tabacum*); and ornamental flowers from the *Petunia *and *Solanum *genera.

Tomato is generally considered to be a model crop plant species, for which many high-quality genetic and genomic resources are available, such as high-density molecular maps [1], many well-characterized near-isogenic lines (NILs), and rich collections of ESTs and full-length cDNAs [2,3]. Potato is the most important crop within the *Solanaceae*, ranking fourth as a world food crop following wheat, maize and rice. Similar resources are available for potato, including an ultra-high density linkage map [4], a collection of phenotype data [5], and a large transcript database [6]. Like most other nightshades, tomato and potato both have a basic chromosome number of twelve, and there is genome-wide colinearity between their genomes [7].

Much effort is currently being invested to sequence the nuclear and organellar genomes of these organisms. The International Tomato Genome Sequencing Project [8] is sequencing the tomato (*S. lycopersicum *cv. Heinz 1706) genome in the context of the family-wide Solanaceae Project (SOL). Rather than sequencing the complete genome, which is approximately 950 Mb [9], only the gene-rich euchromatic regions (estimated at 240 Mb) are being sequenced using a BAC-by-BAC walking approach [10]. The Potato Genome Sequencing Consortium (PGSC) [11] aims to sequence the complete potato (*S. tuberosum*, genotype RH89-039-16) genome of approximately 840 Mb [4] using a similar marker-anchored BAC-by-BAC sequencing strategy.

Both sequencing projects rely heavily on BAC libraries, of which three exist for tomato (HindIII [12], MboI, and EcoRI) and two exist for potato (HindIII and EcoRI). The tomato libraries are available through the SOL Genomics Network (SGN) [13] and the potato libraries will soon by available at through the PGSC [11]. All of these libraries have been end-sequenced to support BAC-by-BAC sequencing and extension, and to provide a base of genome-wide survey sequences to support studies such as the one presented here.

This paper describes the detailed sequence analysis of 310,580 tomato BAC End Sequences (BESs), representing 181.1 Mb (~19%) of the tomato genome, and 128,819 potato BESs, corresponding to 87.0 Mb (~10%) of the potato genome (for an overview of the tomato and potato BES data, see Table 1). This comparative genomics study aims to gain insight into the similarity between the tomato and potato genomes, both on the structural level through repeat and gene content analyses and on the functional level through gene function analyses. Furthermore, we investigate micro-syntenic relationships between these two Solanaceous genomes, and several other sequenced plant genomes. The sequence content of BESs from a particular library is biased by which restriction enzyme was used to make the library. To avoid comparing sequence sets with different biases, tomato-potato comparisons are made only between BESs from libraries made with the same enzyme.

**Table 1:** Overview of tomato and potato BES data

	Number of sequences	Total length	Average length	GC content
**Tomato**	**310,580**	**181,076,819**	**583**	**36.1%**
HBa (HindIII)	144,307	89,649,564	621	35.5%
Eco (EcoRI)	77,141	46,398,406	601	35.2%
Mbo (MboI)	89,132	45,028,849	505	38.3%
**Potato**	**128,819**	**86,972,687**	**675**	**35.6%**
POT (HindIII)	76,930	52,695,698	685	36.0%
PPT (EcoRI)	51,889	34,276,989	661	35.0%

The sequences are subdivided into libraries, which are labeled with a three-letter code, with the corresponding restriction enzyme listed between brackets.

## Results

### Repeat density and categorization

Based on similarity searches of the repeat database, between 13.0% and 22.9% of the nucleotides in the tomato BESs were identified as belonging to a repeat (see Table 2, second through fourth columns). The most common repeat families in the tomato libraries were the Gypsy (5.0 – 11.6%) and Copia (4.2 – 5.3%) classes of retrotransposons. Another prominent class of repeats comprised the ribosomal RNA genes (<0.1 – 8.6%). The tomato Eco (EcoRI) library had the lowest repeat density at 13.0%, which can be attributed to a lower amount of Gypsy retrotransposons (5.0%). The highest repeat content was found in the tomato Mbo (MboI) library (22.9%), more than a third of which (8.6%) consisted of ribosomal RNA genes. Note that, since the repeat detection was based on sequence similarity, different segments in a BES could be assigned to more than one repeat family. As a result, the sum of the repeat content per repeat type can be slightly larger than the total repeat content.

**Table 2:** Classification and distribution of known plant repeats in the BAC end sequences

	Tom. HBa	Tom. Eco	Tom. Mbo	Pot. POT	Pot. PPT
**Class I retrotransposons**	**16.95**	**9.30**	**13.81**	**11.42**	**8.19**
**LTR retrotransposons**	**16.81**	**9.19**	**13.72**	**11.16**	**7.92**
Ty1/Copia	5.25	4.17	4.39	2.55	2.48
Ty3/Gypsy	11.56	5.02	9.33	8.60	5.43
Unclassified	0.00	0.00	0.00	0.01	0.01
**Non-LTR retrotransposons**	**0.14**	**0.11**	**0.09**	**0.26**	**0.27**
LINE	0.09	0.06	0.05	0.15	0.13
SINE	0.05	0.05	0.04	0.11	0.14
**Class II DNA transposons**	**0.64**	**0.66**	**0.49**	**1.03**	**1.23**
En-Spm	0.26	0.26	0.21	0.27	0.27
Harbinger	0.00	0.00	0.00	0.00	0.00
Mariner	0.00	0.00	0.00	0.00	0.00
MuDR	0.07	0.09	0.05	0.10	0.11
Pogo	0.02	0.03	0.02	0.03	0.08
Stowaway	0.02	0.02	0.02	0.01	0.02
TcMar-Stowaway	x	x	x	0.00	0.00
Tourist	x	x	0.00	0.00	x
hAT	0.02	0.04	0.02	0.05	0.19
hAT-Ac	0.01	0.00	0.01	0.01	0.01
hAT-Tip100	0.02	0.02	0.02	0.11	0.10
Unclassified	0.22	0.20	0.14	0.45	0.45
**Satellites**	**0.00**	**0.00**	**0.00**	**0.04**	**0.03**
Centromeric	0.00	x	0.00	0.00	0.00
Subtelomeric	x	x	x	0.00	0.00
Unclassified	0.00	0.00	0.00	0.04	0.03
**Ribosomal genes**	**0.04**	**2.98**	**8.58**	**0.03**	**0.53**
rRNA	0.04	2.98	8.58	0.03	0.53
**Unclassified**	**0.08**	**0.11**	**0.07**	**0.07**	**0.11**
Centromeric	x	x	x	0.00	x
Composite	x	x	x	x	0.00
RC/Helitron	0.08	0.11	0.07	0.06	0.11
Unknown	0.00	0.00	0.00	0.01	0.00
**Total**	**17.66**	**13.01**	**22.91**	**12.54**	**10.02**

Numbers represent percentages of nucleotides that show similarity to a repeat of the indicated category. An 'x' represents the absence of a repeat family; '0.00' indicates that the repeat is present, but at a frequency lower than 0.005 % of the nucleotides in the BESs. Species names have been abbreviated as follows: Tom.: tomato; Pot.: potato.

In contrast to the tomato BESs, only between 10.0% and 12.5% of the nucleotides in the potato BESs showed similarity to known *Magnoliaphytae *repeats (see Table 2, fifth and sixth columns). As in tomato, the majority of the repeats were found in the Gypsy (5.4 – 8.6%) and Copia (2.5 – 2.6%) retrotransposon families, whereas the fraction of ribosomal RNA genes was small (<0.1 – 0.5%). Potato appeared to contain approximately two times as many LINE and SINE elements as tomato (see Table 2), although the absolute percentages were low. Furthermore, a higher percentage of class II DNA transposons was observed in potato (1.0 – 1.2%, versus 0.5 – 0.7% in tomato), the majority of which could not be classified. In agreement with the differences observed between the tomato HBa (HindIII) and Eco libraries, the potato PPT (EcoRI) library had an overall lower repeat content than the POT (HindIII) library, and more specifically, a lower amount of Gypsy retrotransposons (5.4% versus 8.6% in the POT library). The PPT library was also enriched in ribosomal RNA genes in comparison to the POT library (0.5% versus less than 0.1%), just as was found comparing the Eco library to the HBa library in tomato.

Since similarity-based repeat detection can be limited by the size and diversity of the repeat database, a self-comparison of the BESs was performed in order to estimate the redundancy within the BESs. Even with the stringent requirement that at least 50% of a given query sequence match another BES with at least 90% identity, 52.0% of the nucleotides in the tomato BESs had a match to one or more other tomato BESs, and 19.0% matched five or more other BESs. The redundancy in the potato BESs was lower than in tomato; 39.0% of the nucleotides in the potato BESs had a hit to at least one other potato BESs, and 12.9% had a hit to five or more BESs. This difference could not be attributed solely to the larger number of tomato BESs, compared to the number of potato BESs; a self-comparison of the tomato HBa library, which is of approximately the same size as the potato POT and PPT libraries combined, showed that 50.7% of the nucleotides in this library matched at least one other HBa BES, and 16.8% matched five or more other HBa BESs. The percentage of nucleotides in both species that matched five or more other BESs was only slightly higher than the findings from the RepeatMasker analysis (see Table 2), suggesting that the repeat database used in this study was sufficient to detect the majority of highly abundant repeats in these species. These findings also confirm the observation from the similarity-based repeat detection that the tomato BESs are more repetitive than the potato BESs.

### Simple sequence repeats

A total of 28,423 SSRs with a motif length between one and five nt, and a total length of at least 15 nt were detected in the tomato BESs, representing one SSR per 6.4 kb of genomic sequence. The term 'motif length' is used here to describe the length of the motif that is repeated in the SSR; for example, an ATATAT repeat has a motif length of two (with AT being the motif). The most abundant motif length was five nucleotides (11,177 SSRs), followed by motif lengths of two (6,588 SSRs), four (4,596 SSRs), three (4,135 SSRs), and lastly one (1,927 SSRs).

In potato, 19,019 SSRs were found, out of which 3,964 (21%) belonged to class I (i.e., SSRs containing more than 10 motif repeats). Thus, the potato BESs had one SSR per 4.6 kb of genomic sequence, which is higher than that in tomato (one SSR per 6.4 kb). As in tomato, the most abundant motif length in the potato SSRs was five nucleotides (7,922 SSRs). However, the next most abundant length was three (3,941 SSRs), followed by motif lengths of two (3,270 SSRs), four (1,980 SSRs) and one (1,906 SSRs).

**Figure 1:**
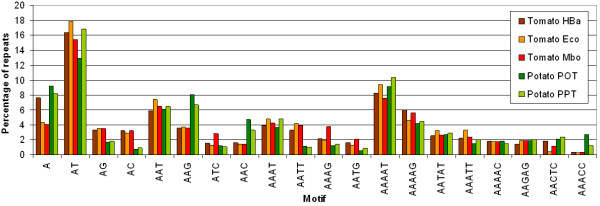
**Distribution of the most abundant SSR motifs in the tomato and potato BESs**. The values on the Y axis represent the fraction of SSRs for each dataset that consist of the motifs listed on the X axis.

Figure 1 shows the distribution of the primary SSR motifs in the tomato and potato BESs, ordered by motif length and relative frequency within the motifs of the same length. The most abundant SSR motifs in both datasets were AT-rich, with the di-nucleotide repeat AT/TA being the most abundant (16.6% of all tomato and 14.7% of all potato SSRs, respectively). Several motifs, such as AG/CT, AC/GT, AATT/AATT and AAAG/CTTT were more frequent in tomato than in potato, whereas other motifs, such as AAG/CTT, AAC/GTT, AACTC/GAGTT and AAACC/GGTTT were found predominantly in potato.

Considering only the class I SSRs, the most abundant SSR motifs in tomato and potato were AT/TA (50.8 and 39.1% of all class I SSRs, respectively) and A/T (25.8 and 42.1%). In tomato, the di-nucleotide motifs AC/GT (6.3%) and AG/CT (5.7%) were the most abundant after these two, whereas in potato the mononucleotide C/G (6.0%) and tri-nucleotide AAT/ATT (4.5%) and AAG/CTT (3.7%) occurred at the second, third and fourth highest frequency, respectively. This suggests that the differences in primary motif frequencies between tomato and potato also hold when considering only class I SSRs.

### Gene content

**Figure 2:**
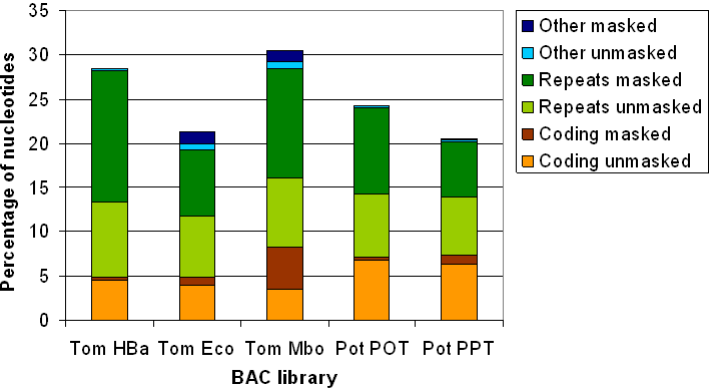
**Percentage of nucleotides in the BESs covered by BLASTX hits to the non-redundant protein database**. The BLAST hits have been divided into three categories ('coding', ' repeats', 'other') based on keyword filtering. Each category has subsequently been divided into 'masked' (i.e., overlapping with repeats identified by RepeatMasker) and 'unmasked' (i.e., no overlap with repeats identified by RepeatMasker) subcategories. Species names have been abbreviated as follows: Tom.: tomato; Pot.: potato.

In the tomato BESs, the percentage of nucleotides that matched by at least one database sequence ranged from 21.3% for the Eco library, to 30.5% for the Mbo library. Figure 2 presents a breakdown of these BLAST hits into three main categories ('coding', 'repeats', and 'other'), based on the keyword filtering described in Materials and Methods. Each category was then subdivided into 'masked' and 'unmasked' subcategories, with 'masked' indicating an overlap with repetitive sequences identified by RepeatMasker, and 'unmasked' indicating a lack of such overlap. In this way, the BLAST and RepeatMasker results were combined in order to generate the best possible estimation of the percentage of putative protein-coding nucleotides in the BESs. The 'coding' category represents the percentage of nucleotides that matched one or more database sequences, and were not identified as repetitive by the keyword filtering. After removing the overlap with repeats identified by RepeatMasker, the percentage of coding nucleotides in the three libraries ranged from 3.5% for the Mbo library to 4.6% for the HBa library (the 'coding unmasked' category in Figure 2). The Mbo library had the highest percentage of the three libraries in the 'coding masked' category, which is likely the result of the high number of ribosomal repeat sequences in this library that have escaped the keyword filtering. The 'repeats' category contains the BLAST matches to transposon and other repeat related sequences. In all three libraries, there was a considerable fraction of nucleotides that the keyword filtering assigned to the 'repeats' category but that did not overlap with the repeats identified by RepeatMasker (i.e. the 'repeats unmasked' category). This fraction ranged from 6.9% in the Eco library to 8.4% in the HBa library and may represent a combination of repeats that were missed by RepeatMasker and true protein-coding genes that were miss-classified by the keyword filtering. The final category in Figure 2, 'other', represents all non-transposon-related repetitive sequences that were identified by the keyword filtering (all keyword terms other than "Transposon terms" from Additional File 1).

In the potato POT and PPT libraries, 24.3 and 20.5% of the nucleotides matched the protein database, respectively. While these numbers were slightly lower than those for the tomato HBa and Eco libraries (28.5 and 21.3%, respectively), the percentage of nucleotides assigned to the 'coding' category (6.8 and 6.3%) was larger than those of the corresponding tomato libraries (4.6 and 3.9%), suggesting that potato may have a larger gene repertoire than tomato. Furthermore, the number of transposon regions and other repeat-related regions that was found in this comparison to the protein database was more than 1.5-fold higher for tomato than for potato. This is consistent with the difference in transposon content that was found in the repeat analysis.

**Figure 3:**
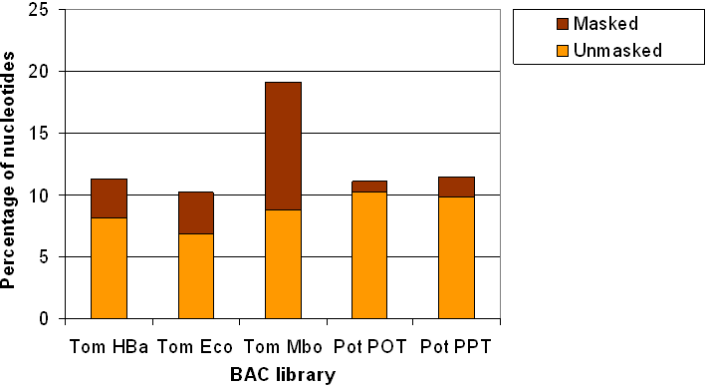
**Percentage of nucleotides in the BESs covered by BLASTN hits to the species-specific transcript databases**. The BLAST hits have been divided into 'masked' (i.e., overlapping with repeats identified by RepeatMasker) and 'unmasked' (i.e., no overlap with repeats identified by RepeatMasker) categories. Species names have been abbreviated as follows: Tom.: tomato; Pot.: potato.

Figure 3 shows the results of the BLASTN comparison of the BESs to species-specific EST databases. The matches were divided into two categories, 'masked' and 'unmasked'. The 'masked' category contains the nucleotides that had a match in the EST database, but were found to be repetitive in the RepeatMasker analysis; the 'unmasked' category contains the nucleotides that did not overlap with repeats. In the tomato libraries, between 10.2 and 19.1% of the nucleotides matched one or more tomato EST sequences. The Mbo library had the highest EST coverage (19.1%), but more than half of these matches (10.3%) were 'masked'. The percentage of nucleotides in the 'unmasked' category ranged from 6.8% in the Eco library to 8.8% in the Mbo library.

For the potato BESs, 11.1% (POT) and 11.5% (PPT) of the nucleotides had match in the potato EST database, which is in fairly good agreement with the tomato HBa and Eco comparisons versus the tomato database (11.3 and 10.2%, respectively; see also Figure 3). Fewer matches in the potato BESs were 'masked' than in tomato, confirming the observation from the BLASTX comparison to the protein database that the potato BESs have more protein coding nucleotides and lower repeat content.

### Functional annotation

A total of 30,335 GO terms, out of which 585 unique terms, were assigned to the tomato HBa BESs based matches in the Pfam database (see Additional Files 2, 3, 4, 5 for an overview of all GO terms and their corresponding frequencies in the tomato and potato BESs). Although there were more than half as many Eco BESs as HBa BESs, only 7,647 GO terms (403 unique terms) were assigned to them. In potato, 17,060 terms (544 unique terms) were assigned to the POT library, whereas only 9,312 terms (419 unique terms) were assigned to the PPT library. Comparing the GO annotations of tomato to those of potato (for libraries generated with the same restriction enzyme) resulted in 18 significantly overrepresented terms between the HindIII digested libraries (seven in tomato HBa, and eleven in potato POT; P values are found in Additional File 3) and nine significantly overrepresented terms between the EcoRI digested libraries (seven in tomato Eco, and two in potato PPT; P values are found in Additional File 2).

In both species, many of the terms that were overrepresented in the HindIII libraries compared to their EcoRI counterparts were related to retrotransposon activity, such as DNA binding (GO:0003677), DNA integration (GO:0015074), RNA-directed DNA polymerase activity (GO:0005634), and chromatin-related terms (GO:0000785, GO:0003682, GO:0006333). Furthermore, many of these transposon-related terms were significantly overrepresented in tomato, compared to potato (P value < 10^-4^; individual P values are found in Additional Files 2 and 3). This is consistent with the findings from the RepeatMasker and BLAST analyses discussed above. Surprisingly, some terms that were overrepresented in both the EcoRI digested libraries could be linked to transcription factor genes. In tomato, zinc ion binding (GO:0008270), DNA-dependent regulation of transcription (GO:0006355), and transcription factor activity (GO:0003700) were overrepresented in the Eco library. The potato PPT library was enriched for zinc ion binding (GO:0008270), nucleic acid binding (GO:0003676), and transcription factor activity (GO:0003700).

Analysis of the protein families identified by PANTHER revealed similar trends for the number of matches, both within and between the tomato and potato libraries (see Additional Files 6, 7, 8, 9 for an overview of all PANTHER terms and their corresponding frequencies in the tomato and potato BESs). In tomato, 1,064 distinct families were found in the HBa BESs for a total of 28,984 hits, and 8,226 hits representing 654 families were found in the Eco BESs. Analysis of the potato POT library revealed 951 distinct PANTHER families for a total of 13,821 hits; however, only 6,926 hits to 716 families were found in the PPT BESs. Two and three PANTHER families were found to be overrepresented in the tomato HBa and Eco libraries, compared to eleven and five overrepresented families in the potato POT and PPT libraries, respectively.

Consistent with the greater abundance of Gypsy retrotransposons in the HindIII libraries of both tomato and potato, the GAG/POL/ENV polyprotein (PTHR10178) PANTHER family was found to be overrepresented in both HindIII libraries, compared to the corresponding EcoRI libraries. Furthermore, the GAG-POL-related retrotransposon (PTHR11439) PANTHER family was relatively more abundant in the EcoRI libraries, which also agrees with the difference in the Gypsy:Copia ratio between the HindIII and EcoRI libraries (see also Table 2). Both of these retrotransposon-related terms were found to be significantly (P value < 10^-4^; individual P values are found in Additional Files 6 and 7) overrepresented in tomato when compared to potato. In the tomato Eco library, transcription-factor related terms such as zinc finger CCHC domain contain protein (PTHR23002), zinc finger protein (PTHR11389) and MADS box protein (PTHR11945) were significantly overrepresented (P values 4.0*10^-13^, 7.8*10^-7^, and 1.5*10^-6^, respectively), confirming the results from the GO analysis. No transcription-factor related PANTHER families were significantly overrepresented in the potato PPT library.

Between tomato and potato, the majority of the overrepresented terms in potato corresponded to important biological and biochemical processes. For example, zinc finger CCHC domain containing proteins (PTHR23002) and general transcription factor 2-related zinc finger proteins (PTHR11697) occurred with a significantly (P value 2.2*10^-16 ^for both) higher frequency in potato POT than in tomato HBa; the latter was also overrepresented in the potato PPT library. This was also reflected in the GO annotation through terms such as nucleic acid binding (GO:0003676) and zinc ion binding (GO:0008270). The overrepresentation of these terms relative to tomato suggests an expansion of transcription factors or other genes for DNA binding proteins in the potato genome.

Another example is the cytochrome P450 superfamily (PTHR19383), which was also found in the GO analysis through terms such as iron ion binding (GO:0005506) and mono-oxygenase activity (GO:0004497). Cytochrome P450 proteins play important roles in the biosynthesis of secondary metabolites, and the overrepresentation of these proteins in potato could indicate an expanded network of pathways that synthesize secondary metabolites in potato.

A final example involves the large family of plant-type serine-threonine protein kinases (PTHR23258), which are known to play important roles in disease resistance in various plant species (for example, the Pto gene in tomato [14]). In the PANTHER database, this family consists of 104 different subfamilies, 71 of which were found in the tomato and potato BESs. Out of these 71 subfamilies, 15 were found only in tomato, and five were unique to potato. Most of the subfamilies that were found in both species were overrepresented in potato, such as LRR receptor-like kinases (PTHR23258:SF462) and LRR transmembrane kinases (PTHR23258:SF474). Several subfamilies occurred at a higher frequency in tomato, including serine/threonine specific receptor-like protein kinases (PTHR23258:SF416) and Pto-like kinases (PTHR23258:SF418). Thus, while the complement of serine-threonine protein kinases in potato exceeds that of tomato, several of the subfamilies have expanded specifically in tomato. This may reflect an adaptation for resistance to different pathogens, or a difference in the dominant mechanism of pathogen resistance between these species.

### Comparative genome mapping

Out of the 135,842 pairs of tomato BESs that were compared to the *A. thaliana *genome, 15,283 pairs had one or more matches. These matches were divided into five categories, as is shown in the last five columns of Table 3. The 'single end' category represents the BAC end pairs from which only one of the two sequences had a match to the *A. thaliana *genome, and contained the majority of the matches (10,191). Paired end matches, in which the BESs from the same BAC each had a match to a different chromosome, were assigned to the 'non-linear' category. The 'gapped' category contained 4,836 BAC end pairs that matched to the same *A. thaliana *chromosome with a distance between the paired matches that was either smaller than 50 kb or larger than 500 kb. The final two categories represented the BACs from which both end sequences were matched to the genome within a distance of 50 to 500 kb of each other, either in the correct orientation with respect to each other ('colinear'), or rearranged with respect to each other ('rearranged'). Out of the 4,840 tomato BES pairs that hit to the same *A. thaliana *chromosome, three pairs fell into the 'colinear' category, and one pair fell into the 'rearranged' category, suggesting the presence of four putative micro-syntenic regions between tomato and *A. thaliana*.

**Table 3:** BLASTN hits between the tomato and potato BESs, and the A. thaliana genome

	No hit	Single end	Non-linear	Gapped	Colinear	Rearranged
**Tomato**	**120,559**	**10,191**	**252**	**4,836**	**3**	**1**
HBa	57,489	5,469	159	50	1	1
Eco	30,529	1,655	33	1,279	2	0
Mbo	32,541	3,067	60	3,507	0	0
**Potato**	**51,361**	**4,102**	**82**	**115**	**1**	**1**
POT	31,568	2,718	57	18	1	0
PPT	19,793	1,384	25	97	0	1

Potato had 55,662 pairs of BESs, out of which 117 pairs were mapped to the *A. thaliana *genome, with both BESs of the pair matching the same chromosome. Two potato BACs displayed putative microsynteny based on the end sequence matches, one of which was colinear, whereas the other represented a possible rearrangement. In comparison to tomato, potato had very few BACs that fell into the 'gapped' category, although the smaller PPT library had more than five times as many sequences in this category as the POT library. Interestingly, the large majority of the tomato BACs that fell into this category was from the Eco and Mbo libraries (1,279 and 3,507, respectively). The EcoRI and MboI digested libraries were found to contain a high fraction of ribosomal RNA genes in the RepeatMasker analysis, and indeed more than 80% of the sequences from these libraries that fell into the 'gapped' category contained ribosomal RNA genes.

Repeating the same analysis against the *P. trichocarpa *genome, only 708 of the tomato BES pairs matched with both ends to the same chromosome (the sum of the last three columns in Table 4). It should be noted here that *P. trichocarpa *has both a larger number of chromosomes than *A. thaliana *(19 versus 5) and approximately twenty-two thousand additional contig sequences that have not yet been integrated into the chromosome pseudomolecules. Based on these numbers alone, one would expect a smaller number of paired BESs to map to the same chromosome or contig sequence. Even so, *P. trichocarpa *displayed more regions of micro-synteny with tomato than *A. thaliana*: 73 pairs of BESs mapped within a distance between 50 and 500 kb of the other BES in the pair. More than two-thirds of these matches (51, the 'colinear' category in Table 4) showed colinearity between tomato and *P. trichocarpa*, whereas the remaining 22 hits represented rearrangements in their respective regions of micro-synteny.

**Table 4:** BLASTN hits between the tomato and potato BESs, and the P. trichocarpa genome

	No hit	Single end	Non-linear	Gapped	Colinear	Rearranged
**Tomato**	**110,633**	**18,904**	**5,597**	**635**	**51**	**22**
HBa	52,083	10,297	666	68	38	17
Eco	28,630	3,341	1,174	344	6	3
Mbo	29,920	5,266	3,757	223	7	2
**Potato**	**46,189**	**8,844**	**554**	**34**	**24**	**17**
POT	28,116	5,899	300	19	17	11
PPT	18,073	2,945	254	15	7	6

Consistent with the difference between the tomato – *A. thaliana *and tomato – *P. trichocarpa *mappings, a smaller number of potato BES pairs (75) could be mapped with both ends to the same chromosome in *P. trichocarpa*, than in *A. thaliana*. Of these, there were 41 regions of potential microsynteny, out of which 24 were colinear. Compared to tomato, the 'non-linear' and to a lesser extent the 'gapped' categories were underrepresented in potato. Again these differences seem to originate from the fact that many of the BESs in the Eco and Mbo libraries contain ribosomal RNA genes. The majority of these sequences fell into the 'non-linear' category in the *P. trichocarpa *comparison, rather than the 'gapped' category as was the case with *A. thaliana*, due to the ribosomal RNA genes being contained in some of the unassembled contig sequences rather than in the chromosomal pseudomolecules.

## Discussion

### Sequence properties

Based on the differences between the libraries in both tomato and potato, it seems unlikely that any of these partial digestion-based libraries represents an unbiased cross section of the genome. For example, in tomato the Mbo library has a higher GC percentage than the HBa and Eco libraries. This difference is likely caused by the length and GC content of the restriction sites that were targeted in the digestion of the genome: both the HindIII and EcoRI sites (AAGCTT and GAATTC, respectively) have a length of six nucleotides and a GC content of 33.3%, whereas the MboI site (GATC) has a length of four nucleotides and a GC content of 50%. The consequences of this are clearly visible in the results of the gene and repeat content analyses presented in this paper: results differ markedly among libraries made with different enzymes. However, we think it reasonable to assume that tomato and potato libraries derived from digestion with the same restriction enzyme would have similar sequence bias. Using this assumption, we strive to minimize any effect of sequence bias on our results by maintaining logical separation of BESs from different libraries, and only directly comparing data for BESs from libraries constructed with the same restriction enzymes.

The tomato BESs (and specifically the Mbo BESs) are shorter than the potato BESs on average. The difference in average sequence length between the tomato HindIII and EcoRI libraries and their potato counterparts is approximately 60 nt for both libraries and is most likely the result of a difference in sequencing quality and equipment. However, we think it reasonable to assume that a difference in sequence length on this scale would not influence the results of the similarity-based analyses that have been performed in this study.

### Repeat density and categorization

Both the tomato and potato libraries vary in total repeat content and in ratios between repeat types. For example, ribosomal DNA sequences are overrepresented in the tomato Mbo and Eco, and the potato PPT libraries, relative to the tomato HBa and potato POT library, respectively. This phenomenon was also observed in a study of *Zea mays *BESs [15], where it was attributed to the presence of many MboI sites in the *Z. mays *ribosomal DNA cluster, compared to one EcoRI site, and no HindIII sites. By similar reasoning, the under-representation of Gypsy retrotransposons in the Eco and PPT libraries might result from a lower frequency of EcoRI sites in this element compared to HindIII and MboI sites.

The discrepancy between the repeats identified by RepeatMasker (Table 2) and BLASTX (Figure 2) indicates the need for tomato- and potato-specific repeat databases. A repeat database had previously been generated from the tomato BESs (L. Mueller, unpublished data), however comparing the tomato BESs to this database using RepeatMasker resulted in approximately 60% of the tomato BESs being annotated as repetitive (data not shown). The majority of these repeats could however not be assigned to a known repeat family. Thus, while the findings in this paper may present an underestimation of the actual repeat content of the tomato and potato BESs, the findings from the RepeatMasker and BLASTX analyses both clearly suggest a higher repeat content in the tomato BESs than in the potato BESs.

A correlation between genome size and retrotransposon content has previously been identified in the *Brassicaceae *[16]. There, it was found that the retrotransposon content increases with genome size, from approximately 7 to 10% in *A. thaliana *(genome size 125 Mb), to 14% in *Brassica rapa *(genome size 530 Mb), to 20% in *B. olacerea *(genome size 700 Mb). Comparing this to cereal crops such as *Oryza sativa *(genome size 430 Mb, 35% retrotransposons [17] and *Z. mays *(genome size 2,365 Mb, 56% retrotransposons [15]) suggests that while the actual retrotransposon content in cereals is higher than in *Brassicaceae*, the correlation with genome size may be universally present in plants. The data presented in this research indicate that genome expansion in the *Solanaceae *is also associated with retrotransposon amplification; potato (genome size 840 Mb) has an estimated retrotransposon content between 8.2 (PPT) and 11.4% (POT), whereas that of tomato (genome size 950 Mb) is notably higher (9.3% for the Eco library, and 17.0% for the HBa library).

The ratio between Gypsy and Copia retrotransposon sequences in the tomato BESs is between 1:1 and 2:1, whereas this ratio in the potato BESs is between 2:1 and 3:1. While this ratio clearly differs within each species between libraries generated with a different restriction enzyme, the difference in ratios between tomato and potato is observed in both the HindIII and the EcoRI digested libraries (see Table 2). In *A. thaliana *[18], *B. rapa *[16], *Carica papaya *[19] and *Z. mays *[15], this ratio is approximately 1:1. The tomato and potato genomes appear more similar to the *O. sativa *genome in this respect, where the Gypsy to Copia ratio was found to be around 2:1 [17]. The difference in the Gypsy:Copia ratio between tomato and potato suggests that the retrotransposon amplification associated with the genome expansion in tomato is predominantly the result of additional Copia copies.

### Simple sequence repeats

The most abundant SSRs in all size categories for both tomato and potato were AT-rich. This is consistent with findings in other plant species, such as *A. thaliana *[20], *B. rapa *[16], *C. papaya *[19], *Glycine max *[21], and *Musa acuminata *[22]. In both potato and tomato, penta-nucleotide repeats are the most common form of SSRs, and AAAAT is the predominant repeat motif. This is in sharp contrast to previously studied plant species, in which di- and penta-nucleotide repeats generally occur least frequently [23]. In many plant species, such as *A. thaliana*, *B. rapa *[16], and *O. sativa *[24,25], tri-nucleotide repeats are the most abundant microsatellites. However, BES analysis of *C. papaya *[19], *G. max *[21] and *M. acuminata *[22] suggests that di-nucleotide repeats are more common in these plant species. Thus, both tomato and potato display a unique distribution of microsatellite frequencies compared to other studied plant species.

The tomato BESs have a higher fraction of di- and tetra-nucleotide repeats compared to the potato BESs. This may be because one or more of the tomato BAC end libraries are enriched for BACs that are derived from centromeric regions in the tomato genome, as these regions have previously been found to be enriched for long, class I di- and tetra-nucleotide repeats [26]. However, the relative enrichment for di- and tetra-nucleotide repeats in tomato compared to potato is observed in all three tomato libraries; this would only be compatible with the hypothesis of enrichment for centromeric regions if these regions contain more HindIII, EcoRI and MboI sites than average for the tomato genome.

### Gene content

After repeat masking and keyword filtering, the percentage of nucleotides in the potato POT and PPT BESs that have a match in the non-redundant protein database is 1.5- to 1.6-fold that of the tomato HBa and Eco BESs, respectively. Both the percentage of nucleotides and the number of BESs having a hit to the protein database after repeat masking and keyword filtering are higher in potato (13.8% in the POT library; 12.9% in the PPT library) than in tomato (8.7% in the HBa library; 7.9% in the Eco library), supporting the hypothesis that potato has more putative protein-coding regions than tomato. In the BLASTN comparison of the BESs to the ESTs, a similar discrepancy between potato and tomato was observed, with potato having a 1.3- to 1.4-fold higher EST coverage than tomato. Furthermore, cross-comparisons of the tomato BESs to the potato ESTs and vice versa confirmed that the difference in EST coverage of the BESs was not caused by a difference in number of unique transcripts between the tomato and potato EST collections (data not shown). The difference between the BLAST comparisons to the protein and transcript databases may be attributed to the presence of full-length cDNA sequences in the tomato transcript data, whereas these are not present in the potato data, resulting in an overrepresentation in the tomato BESs for the interior regions of coding sequences. Even if one assumes that this more conservative lower bound is correct, the results still suggest that potato has a larger gene repertoire than tomato since the tomato genome is only approximately 1.1 times larger than the potato genome.

In both tomato and potato, a smaller percentage of nucleotides show similarity to the EST database than to the protein database, while the percentage of non-repetitive coding sequence in the EST database comparison (the 'unmasked' category in Figure 3) is higher than that in the protein database comparison (the 'coding unmasked' category in Figure 2). Surprisingly, the majority of the matches to the protein and transcript databases do not overlap. For example, in the tomato HBa library, 8.1% and 4.6% of the nucleotides have a match in the EST and protein databases, respectively, while only 1.6% have a match in both. Similarly, for the potato POT library, only 2.5% of the nucleotides have a match in both the transcript and protein sequences, whereas the individual percentages of nucleotides that have a match in these databases are 10.2% and 6.8%, respectively. On one hand, the matches to the EST databases that do not overlap with matches to the protein database may represent unique, taxon- or species-specific protein-coding genes that are not represented in the non-redundant protein database, or transcribed but untranslated regions in these genomes. On the other hand, matches to the protein database that do not overlap with matches in the EST database may indicate either the presence of genes that were not sufficiently expressed in the tissues under the conditions that were sampled during EST library construction, or mis-annotated or otherwise incorrect sequences in the protein database.

The EST data likely provides the most reliable sampling of the true protein coding regions in these genomes, since it is based on experimental data that contain species-specific sequences not available in the protein database. Due to the selection for poly-A tails that is normally used in the construction of EST libraries, the number of non-protein coding transcripts will be relatively small. Taking the nucleotides from the HBa and Eco libraries that match ESTs and do not overlap with repeats as a measure of coding sequences, the tomato genome (950 Mb) is estimated to contain between 64.8 and 77.1 Mb of coding regions. Similarly, assuming a genome size of 840 Mb, the total coding region length for potato would be between 82.5 and 85.4 Mb. These numbers set lower bounds on the estimated coding content of these genomes, as the EST data is unlikely to represent the full complement of full-length protein-coding sequences in these genomes.

Previous estimates put the gene content of tomato at 35,000 genes, based on an analysis of 27,274 UniGenes and 6 BAC sequences [27]. If these 35,000 genes are indeed represented by 71.0 Mb of coding sequence (the average of the estimations for the HBa and Eco libraries), then the average transcript length of tomato would be approximately 2.0 kb. This is longer than the average transcript length in *A. thaliana*, which is 1.2 kb according to the TAIR7 genome annotation [28]. Assuming the same average transcript length, potato (84.0 Mb of coding sequence, averaged over the two libraries) would contain approximately 41,400 genes, or 6,400 more genes than tomato. Since the data presented here are based on similarity-searches on short genomic sequences only, this difference does not necessarily represent a difference in functional genes, but may also reflect a larger proportion of pseudogenes or otherwise non-functional alleles in potato.

### Functional annotation

The results from the GO and PANTHER analysis generally show a similar trend. The tomato BESs have more GO terms and PANTHER families associated to them than the potato BESs do. However, the potato BESs have a larger number of unique terms associated to them. This agrees with the results of the BLASTX comparison to the non-redundant protein database, where it was found that the tomato BESs have a higher overall coverage of BLAST hits, but a lower percentage of putative protein coding regions (see also Figure 2).

In both the GO term and PANTHER family analyses, the majority of the terms occur at a relatively low frequency. For example, in the tomato HBa versus potato POT comparison, only 131 out of the 730 distinct GO terms that were assigned to the BESs occurred ten or more times in at least one of the species. This group of 131 GO terms contained all 18 of the terms that were significantly (P values < 10^-4^) overrepresented in one of the species in this comparison. Moreover, 39 out of these 131 terms were found at least 50 times in at least one species, and this subgroup contained 16 out of the 18 significantly overrepresented terms. Similarly, in the PANTHER family analysis, 119 out of the 1,352 distinct families that were found in the BESs occurred at least ten times in at least one species, out of which 12 families were found at least 50 times. The 119 families that were found at least ten times contained every one of the 13 families that were significantly overrepresented in one of the species; ten of these were counted more than 50 times in at least one species. While only the tomato HBa versus potato POT comparison is shown here, the other comparisons show a similar pattern, indicating that many of the highly abundant GO terms and PANTHER families are significantly overrepresented in either tomato or potato. The majority of these overrepresented terms and families are most abundant in potato, and represent biologically important functions and processes. In tomato, a smaller number of terms and families is overrepresented; these are primarily connected to structural genomic features such as retrotransposons.

The overrepresentation of transposon-related GO terms and PANTHER families in tomato was consistent with the results of the repeat analysis, confirming the observations that tomato is richer in retrotransposons than potato. However, in the PANTHER analysis, reverse transcriptases (PTHR19446) were significantly overrepresented in potato. At first glance, this does not correspond well with the overrepresentation of RNA-directed DNA polymerase activity (GO:0003964) and RNA-dependent DNA replication (GO:0006278) in tomato. However, in both tomato and potato, the large majority of the reverse transcriptases originated from non-LTR retroelements (PTHR19446:SF34), which in fact is consistent with the higher frequency of non-LTR retrotransposons in potato found in the RepeatMasker analysis (see also Table 2).

The cytochrome P450 mono-oxygenases represent a large gene superfamily in plants that are commonly associated with the biosynthesis of secondary metabolites. In *A. thaliana*, at least 272 P450 genes have been found, representing approximately one percent of the gene complement of this species. In *O. sativa*, this family is even larger, with 458 P450 genes identified so far [29]. Not all the P450s in these genomes represent true protein-coding sequences; in *A. thaliana*, 90% of the genes are truly protein coding, compared to 72% in *O. sativa*. In total, 66 distinct families of P450 genes were identified in *A. thaliana *and *O. sativa*, several of which were found to be overrepresented in either of these species. Moreover, some families were present in one, but completely absent in the other species [30]. In the HindIII and EcoRI libraries, 186 and 209 BESs that could be associated with the cytochrome P450 PANTHER family (PTHR19383) were found in tomato and potato, respectively. Since these BAC end sequences represent approximately 14% and 10% of their respective genomes, these data suggest an enormous expansion of P450 genes in the *Solanaceae*. This could be the result of an expansion of specific P450 families, but also of the evolution of species- or family-specific P450s. For example, the allene oxide synthase has currently only been found in Solanaceous species, including tomato and *Petunia inflata *[31]. The overrepresentation of P450s in potato compared to tomato may be another result of species-specific P450 families, but may also indicate expansion of families that are shared between these species.

### Comparative genome mapping

In this study, paired BAC ends have been exploited to detect regions of microsynteny between the Solanaceous species tomato and potato, and the model plant organisms *A. thaliana *and *P. trichocarpa*. Using similar approaches, microsynteny has been observed between *A. thaliana *and *B. rapa *[16]; *C. papaya *and *P. trichocarpa *[19]; and *M. acuminata *and *O. sativa *[22].

A higher number of tomato and potato BACs display microsynteny to *P. trichocarpa*, than to *A. thaliana*. The reduced level of microsynteny between tomato/potato and *A. thaliana *is not likely a difference in evolutionary distances between these species. Both *A. thaliana *and *P. trichocarpa *are part of the rosids clade, whereas tomato and potato belong to the asterids clade. It may be the result of a recent duplication of the *A. thaliana *genome, followed by the loss of roughly 70% of the duplicated genes [32]. Assuming that this loss occurred randomly, the large majority of possible microsyntenic regions that existed before the duplication will have disappeared due to the major local expansions and contractions that would be associated with such a duplication and subsequent loss. This hypothesis is strengthened by the observation that only approximately 1% of 12,000 *A. thaliana *BES pairs could previously be mapped within 300 kb to the *P. trichocarpa *genome, indicating that the organization of these genomes is indeed vastly different [19].

Regions of microsynteny have previously been detected between tomato/potato and *A. thaliana*. A 57 kb region of tomato chromosome 7 containing five genes was shown to be syntenic with a 30 kb region on *A. thaliana *chromosome 1, although the order and orientation of the genes suggested two inversion events [33]. In another study, a 105 kb BAC sequence matched to four different segments on *A. thaliana *chromosomes 2, 3, 4, and 5; however, each of the four regions in *A. thaliana *were shorter than their tomato counterpart [34]. Recently, five microsyntenic blocks were detected between a region on potato chromosome 5 harbouring a QTL for resistance to late blight and root cyst nematodes, and *A. thaliana *chromosomes 1, 3 and 5 [35]. These syntenic blocks spanned between three and seven ORFs, and were interrupted by non-syntenic blocks. In each of these examples, the microsynteny between tomato/potato and *A. thaliana *involves shorter regions on the *A. thaliana *genome than the average tomato and potato BAC sequence length. Furthermore, regions of (micro-)synteny are often detected between coding sequences, whereas the fraction of coding sequences in the tomato and potato BESs is relatively low (< 10%), providing a good explanation for the reduced amount of microsynteny between these species observed here.

Synteny between potato and *A. thaliana *has also been identified on a genome-wide level using a comparative mapping approach. This revealed 90 putative syntenic blocks between potato and *A. thaliana *that cover 41% of the potato genetic map, and 50% of the *A. thaliana *physical map [36]. These syntenic blocks were unevenly distributed over the potato genetic map, and redundant in respect to the number of areas on the *A. thaliana *genome that displayed synteny to most areas on the potato map. The regions of microsynteny between tomato/potato and *A. thaliana *that were found with the BES-based approach described in this study could not be used to confirm or renounce any putative higher-order syntenic regions, due to the relatively short distances between the BAC ends.

Six paired tomato BAC end matches cluster in the 16.0 – 20.2 Mb interval of *P. trichocarpa *chromosome 10. Furthermore, seven pairs of potato BESs map to the partially overlapping interval between 15.2 – 19.4 Mb, indicating the presence of either a number of distinct microsyntenic regions, or possibly a single region of macrosynteny, between the tomato/potato and *P. trichocarpa *genomes. These findings provide an interesting starting point for a detailed comparison between these species in this region, once more tomato and potato genomic sequences become available.

## Conclusion

The large scale analysis of tomato and potato BESs presented in this paper revealed many interesting structural and functional differences between the genomes of these closely related species. We have shown that the tomato genome is not only more repetitive than the potato genome, but that these genomes also differ in their repeat composition, most importantly in the distribution of Gypsy and Copia retrotransposons. In contrast to other studied plant genomes, we have shown that the tomato and potato genomes contain a large number of SSRs with a motif length of five, which may be a unique feature of Solanaceous genomes.

Comparative analysis of the putative protein coding regions in these BESs revealed an enrichment of these regions in the potato genome. Moreover, several protein families were found to be overrepresented in potato compared to tomato, such as cytochrome P450 mono-oxygenases and serine-threonine protein kinases. The P450 superfamily appears to have expanded dramatically in both species compared to *A. thaliana*, suggesting an expanded network of secondary metabolic pathways in the *Solanaceae*.

Both tomato and potato appear to have low microsynteny with *A. thaliana*, which is likely a result of this species' relatively recent genomic rearrangement. A higher degree of synteny was observed with *P. trichocarpa*. Difference in evolutionary distances is not likely to be the reason for this increased microsynteny, since both *A. thaliana *and *P. trichocarpa *are part of the rosids clade, whereas tomato and potato belong to the asterids clade.

Taken together, these findings present a first glimpse into the evolution of Solanaceous genomes, both within the family and relative to other plant species. When the complete genomic sequences of these species become available, whole-genome comparisons and protein- or repeat-family specific studies may shed more light on the intriguing observations made in this paper.

## Methods

### BAC end sequences

Tomato BESs from the HBa (HindIII), Eco (EcoRI) and Mbo (MboI) libraries were obtained from SGN FTP site [13]. For all analyses, the 'screened_and_trimmed' sets (bacends_combined_screened_and_trimmed.v4.seq) were used, in which low-quality regions and vector sequences have been trimmed, and sequences shorter than 150 nt have been removed. Additionally, this file excludes BESs with hits to the mitochondrial genome of *Arabidopsis thaliana *[28] and the chloroplast genome of *N. tabacum *(NCBI GenBank accession NC_001879.2), based on a BLASTN search with an E-value cutoff of 10^-10^. Potato BESs, which have undergone quality and vector clipping, were downloaded from the GSS section of NCBI GenBank [37] using the query "RHPOKEY". All sequences shorter than 150 nt and sequences with BLASTN (blastall 2.2.15) [38] hits to the *A. thaliana *mitochondrial or *N. tabacum *chloroplast genomes with a E-value lower than 10^-10 ^were removed in order to be consistent with the tomato data. Recently, the tomato and potato chloroplast genomes have become available; however, it can be assumed that the *A. thaliana *mitochondrial genome is sufficiently similar to these genomes, and as such additional filtering was not deemed necessary [39,40].

### Repeat density and categorization

Repeats were identified in the tomato and potato BESs through similarity searches to the *Magnoliaphyta *section of the RepBase repeat database (release 2006-10-06) [41], using RepeatMasker 3.1.5 [42] and cross_match 0.990319 [43]. The repeat density was then calculated as the percentage of nucleotides in the BESs that had one or more hits to the repeat database. Classification of repeat families was derived from the annotation in the RepBase database. Redundancy in the BESs was detected with BLASTN (blastall 2.2.15), by comparing the tomato and potato BES data to itself and removing all matches of a sequence to itself. The E-value cutoff was set to 10^-5 ^and BLAST hits were removed if they did not have a minimum coverage of 50% of the query sequence with 90% identity.

### Simple sequence repeats

Microsatellites were detected using a modified version of the Sputnik software [44]. Running parameters were set to return all SSRs spanning at least 15 nucleotides, with a motif length between 1 and 5 (i.e., mono-, di-, tri-, tetra-, and penta-nucleotide repeats), and a minimum score of 8. Microsatellites identified in this manner were divided into two classes; class I, which has 10 or more motif repeats; and class II, which has fewer than 10 motif repeats [21].

### Gene content

The gene content of the BESs was estimated through BLAST searches using blastall 2.2.15. The BESs were compared to the NCBI GenBank non-redundant protein database (release 2007-02-16) [45] using BLASTX, and to the Kazusa KTU2 tomato EST database [46] and the CAB PotatEST potato EST database (January 2007 release) [6] using BLASTN. For all BLAST searches an E-value cutoff of 10^-5 ^was used, and the best five hits were evaluated. Additionally, a 90% identity cutoff was used for the BLASTN searches to the transcript databases.

In order to distinguish between true, putative protein-coding regions, and transposon- or contamination-related regions, the BLAST matches to the non-redundant protein database were filtered based on keyword matches in the BLAST hit descriptions. An overview of the keywords that were used to filter the BLAST results can be found in Additional File 1. In general, these keywords described sequences that show similarity to bacterial contamination, transposon-related, chloroplast, mitochondrial and ribosomal protein sequences. Any BLAST match that was not filtered out by any of the keywords was considered to represent a non-repetitive, protein-coding region.

### Functional annotation

Tomato HBa and Eco, and potato POT and PPT BESs were functionally annotated through comparisons against the Pfam (version 21.0) [47] and PANTHER (version 6.1) [48] protein family databases, using InterProScan 4.3.1 [49]. GO terms from the Pfam annotations, and PANTHER family (but not subfamily) identifiers from the PANTHER annotations, were extracted from the merged output file of InterProScan. For each GO term and PANTHER family, the number of matching tomato and potato BESs was counted; if a single GO term or PANTHER family was assigned to the same sequence multiple times, for example due to multiple open reading frames in the same sequence, it was only counted once.

Subsequently, the counts were compared pairwise using a two-sided Fisher's exact test from the R software suite [50]. Note that GO term annotations are not always assigned independently of each other (as is required by Fisher's exact test), meaning that some terms often or exclusively occur together as they both describe different aspects of a single biological process or function. However, for simplicity, these higher order dependencies between GO terms are ignored, which may lead to an overestimation of the number of distinct overrepresented terms. Additionally, to mitigate error caused by differences in bias between libraries made with different restriction enzymes, direct inter-species comparisons are made only between BESs from libraries made with the same restriction enzyme. Lastly, the null hypothesis here is that there is no difference in abundance for a GO term or PANTHER family between the tomato and potato BESs, whereas the alternative hypothesis indicates a difference. A conservative P value cut-off of 10^-4 ^was selected to distinguish significant differences from non-significant differences.

### Comparative genome mapping

To determine potential areas of microsynteny between the Solanaceous species studied here and dicot model plants, paired BESs were selected and mapped to the *A. thaliana *and *Populus trichocarpa *genome sequences with BLASTN alignments. Paired end sequences were available for 135,842 tomato BACs (63,169 HBa, 33,498 Eco and 39,175 Mbo) and 55,662 potato (34,362 POT and 21,300 PPT) BACs. Whole genome sequences of *A. thaliana *and *P. trichocarpa *were downloaded from TAIR [28] and JGI [51], respectively. The *P. thrichocarpa *genome sequence used in this study was not finished, but rather consisted of a pseudomolecule sequence for each of the 19 chromosomes plus an additional 177,7 Mb in 21,993 contig sequences.

For each BES, only the best match to the respective genome sequence with an E-value lower than 10^-5 ^was evaluated, and the hit was rejected if the distance between subsequent HSPs was larger than 2000 nt. A BAC was considered to have microsynteny to the target genome if both ends mapped within a distance of between 50 and 500 kb of one another. When both ends were oriented properly with respect to each other, the region was considered colinear; otherwise, the region was considered to be rearranged between the two species.

## List of abbreviations

BAC = Bacterial Artificial Chromosome; BES = BAC End Sequence; Eco = Tomato EcoRI digested BAC library; EST = Expressed Sequence Tag; GO = Gene Ontology; HBa = Tomato HindIII digested BAC library; HSP = High-scoring Segment Pair; kb = kilobases; Mb = Megabases; Mbo = Tomato MboI digested BAC library; nt = nucleotides; POT = Potato HindIII digested BAC library; PPT = Potato EcoRI digested BAC library; SSR = Simple Sequence Repeat.

## Authors' contributions

ED conceived the study, performed all computational analyses and drafted the manuscript. LM, RB, JG were responsible for the BAC end sequencing of tomato and together with RV contributed to the interpretation of the computational analyses and provided feedback on the final draft version of the manuscript. WS and RvH participated in the design and coordination of the study, and helped to draft the manuscript. All authors read and approved the final manuscript.

## Supplementary Material

Additional file 1 This file describes the keyword filtering that has been applied after the BLASTX searches to the non-redundant protein database, in order to distinguish between true putative protein-coding regions, and repetitive and/or contamination-related sequences. Click here for file

Additional file 2 This file describes the Gene Ontology terms found in the InterProScan analysis of the tomato and potato EcoRI digested BAC end sequences. The columns in this Table describe the GO term, the number of BAC end sequences in the tomato Eco and potato PPT library that had this term assigned to them, and the P value of Fisher's exact test for the difference of relative abundance of this GO term between these two libraries. A P value lower than 10^-4 ^indicates a significant difference in the abundance of a GO term between these libraries. Click here for file

Additional file 3 This file describes the Gene Ontology terms found in the InterProScan analysis of the tomato and potato HindIII digested BAC end sequences. The columns in this Table describe the GO term, the number of BAC end sequences in the tomato HBa and potato POT library that had this term assigned to them, and the P value of Fisher's exact test for the difference of relative abundance of this GO term between these two libraries. A P value lower than 10^-4 ^indicates a significant difference in the abundance of a GO term between these libraries. Click here for file

Additional file 4 This file describes the Gene Ontology terms found in the InterProScan analysis of the potato HindIII and EcoRI digested BAC end sequences. The columns in this Table describe the GO term, the number of BAC end sequences in the potato POT and PPT library that had this term assigned to them, and the P value of Fisher's exact test for the difference of relative abundance of this GO term between these two libraries. A P value lower than 10^-4 ^indicates a significant difference in the abundance of a GO term between these libraries. Click here for file

Additional file 5 This file describes the Gene Ontology terms found in the InterProScan analysis of the tomato HindIII and EcoRI digested BAC end sequences. The columns in this Table describe the GO term, the number of BAC end sequences in the tomato HBa and Eco library that had this term assigned to them, and the P value of Fisher's exact test for the difference of relative abundance of this GO term between these two libraries. A P value lower than 10^-4 ^indicates a significant difference in the abundance of a GO term between these libraries. Click here for file

Additional file 6 This file describes the PANTHER families found in the InterProScan analysis of the tomato and potato EcoRI digested BAC end sequences. The columns in this Table describe the PANTHER family, the number of BAC end sequences in the tomato Eco and potato PPT library that had this term assigned to them, and the P value of Fisher's exact test for the difference of relative abundance of this GO term between these two libraries. A P value lower than 10^-4 ^indicates a significant difference in the abundance of a GO term between these libraries. Click here for file

Additional file 7 This file describes the PANTHER families found in the InterProScan analysis of the tomato and potato HindIII digested BAC end sequences. The columns in this Table describe the PANTHER family, the number of BAC end sequences in the tomato HBa and potato POT library that had this term assigned to them, and the P value of Fisher's exact test for the difference of relative abundance of this GO term between these two libraries. A P value lower than 10^-4 ^indicates a significant difference in the abundance of a GO term between these libraries. Click here for file

Additional file 8 This file describes the PANTHER families found in the InterProScan analysis of the potato HindIII and EcoRI digested BAC end sequences. The columns in this Table describe the PANTHER family, the number of BAC end sequences in the potato POT and PPT library that had this term assigned to them, and the P value of Fisher's exact test for the difference of relative abundance of this GO term between these two libraries. A P value lower than 10^-4 ^indicates a significant difference in the abundance of a GO term between these libraries. Click here for file

Additional file 9 This file describes the PANTHER families found in the InterProScan analysis of the tomato HindIII and EcoRI digested BAC end sequences. The columns in this Table describe the PANTHER family, the number of BAC end sequences in the tomato HBa and Eco library that had this term assigned to them, and the P value of Fisher's exact test for the difference of relative abundance of this GO term between these two libraries. A P value lower than 10^-4 ^indicates a significant difference in the abundance of a GO term between these libraries. Click here for file

## References

[B1] Tanksley SD, Ganal MW, Prince JP, de Vicente MC, Bonierbale MW, Broun P, Fulton TM, Giovannoni JJ, Grandillo S, Martin GB, Messeguer R, Miller JC, Miller L, Paterson AH, Pineda O, Röder MS, Wing RA, Wu W, Young ND (1992). High density molecular linkage maps of the tomato and potato genomes. Genetics.

[B2] D'Agostino N, Aversano M, Frusciante L, Chiusano ML (2007). TomatEST database: in silico exploitation of EST data to explore expression patterns in tomato species. Nucleic Acids Res.

[B3] Yano K, Watanabe M, Yamamoto N, Tsugane T, Aoki K, Sakurai N, Shibata D (2006). MiBASE: A database of a miniature tomato cultivar Micro-Tom. Plant Biotechnology.

[B4] Van Os H, Andrzejewski S, Bakker E, Barrena I, Bryan GJ, Caromel B, Ghareeb B, Isidore E, De Jong W, Van Koert P, Lefebvre V, Milbourne D, Ritter E, Rouppe van der Voort JNAM, Rousselle-Bourgeois F, Van Vliet J, Waugh R, Visser RGF, Bakker J, Van Eck HJ (2006). Construction of a 10,000-Marker Ultradense Genetic Recombination Map of Potato: Providing a Framework for Accelerated Gene Isolation and a Genomewide Physical Map. Genetics.

[B5] Wageningen UR Plant Breeding CBSG Potato & Tomato Genomics Database. http://potatodbase.dpw.wau.nl/.

[B6] PotatEST DB. http://biosrv.cab.unina.it/potatestdb/.

[B7] Bonierbale MW, Plaisted RL, Tanksley SD (1988). RFLP Maps Based on a Common Set of Clones Reveal Modes of Chromosomal Evolution in Potato and Tomato. Genetics.

[B8] Mueller LA, Tanksley SD, Giovannoni JJ, Van Eck J, Stack S, Choi D, Kim BD, Chen M, Cheng Z, Li C, Ling H, Xue Y, Seymour G, Bishop G, Bryan G, Sharma R, Khurana J, Tyagi A, Chattopadhyay D, Singh NK, Stiekema W, Lindhout P, Jesse T, Klein Lankhorst R, Bouzayen M, Shibata D, Tabata S, Granell A, Botella MA, Giuliano G, Frusciante L, Causse M, Zamir D (2005). The Tomato Sequencing Project, the first cornerstone of the International Solanaceae Project (SOL). Comparative and Functional Genomics.

[B9] Arumuganathan K, Earle ED (1991). Nuclear DNA content of some important plant species. Plant Mol Biol.

[B10] Mueller LA, Solow TH, Taylor N, Skwarecki B, Buels R, Binns J, Lin C, Wright MH, Ahrens R, Wang Y, Herbst EV, Keyder ER, Menda N, Zamir D, Tanksley SD (2005). The SOL Genomics Network: a comparative resource for Solanaceae biology and beyond. Plant Physiol.

[B11] Potato Genome Sequencing Consortium. http://www.potatogenome.net/.

[B12] Budiman MA, Mao L, Wood TC, Wing RA (2000). A Deep-Coverage Tomato BAC Library and Prospects Toward Development of an STC Framework for Genome Sequencing. Genome Res.

[B13] SOL Genomics Network. http://sgn.cornell.edu/.

[B14] Martin GB, Brommonschenkel SH, Chunwongse J, Frary A, Ganal MW, Spivey R, Wu T, Earle ED, Tanksley SD (1993). Map-based cloning of a protein kinase gene conferring disease resistance in tomato. Science.

[B15] Messing J, Bharti AK, Karlowski WM, Gundlach H, Kim HR, Yu Y, Wei F, Fuks G, Soderlund CA, Mayer KF, Wing RA (2004). Sequence composition and genome organization of maize. Proc Natl Acad Sci U S A.

[B16] Hong CP, Plaha P, Koo DH, Yang TJ, Choi SR, Lee YK, Uhm T, Bang JW, Edwards D, Bancroft I, Park BS, Lee J, Lim YP (2006). A Survey of the Brassica rapa genome by BAC-end sequence analysis and comparison with Arabidopsis thaliana. Mol Cells.

[B17] International Rice Genome Sequencing Project (2005). The map-based sequence of the rice genome. Nature.

[B18] Arabidopsis Genome Initiative (2000). Analysis of the genome sequence of the flowering plant Arabidopsis thaliana. Nature.

[B19] Lai CW, Yu Q, Hou S, Skelton RL, Jones MR, Lewis KL, Murray J, Eustice M, Guan P, Agbayani R, Moore PH, Ming R, Presting GG (2006). Analysis of papaya BAC end sequences reveals first insights into the organization of a fruit tree genome. Mol Genet Genomics.

[B20] Katti MV, Ranjekar PK, Gupta VS (2001). Differential distribution of simple sequence repeats in eukaryotic genome sequences. Mol Biol Evol.

[B21] Shultz JL, Kazi S, Bashir R, Afzal JA, Lightfoot DA (2007). The development of BAC-end sequence-based microsatellite markers and placement in the physical and genetic maps of soybean. Theoretical and Applied Genetics.

[B22] Cheung F, Town CD (2007). A BAC end view of the Musa acuminata genome. BMC Plant Biol.

[B23] Mun JH, Kim DJ, Choi HK, Gish J, Debellé F, Mudge J, Denny R, Endré G, Saurat O, Dudez AM, Kiss GB, Roe B, Young ND, Cook DR (2006). Distribution of microsatellites in the genome of Medicago truncatula: a resource of genetic markers that integrate genetic and physical maps. Genetics.

[B24] Goff SA, Ricke D, Lan TH, Presting G, Wang R, Dunn M, Glazebrook J, Sessions A, Oeller P, Varma H, Hadley D, Hutchison D, Martin C, Katagiri F, Lange BM, Moughamer T, Xia Y, Budworth P, Zhong J, Miguel T, Paszkowski U, Zhang S, Colbert M, Sun WL, Chen L, Cooper B, Park S, Wood TC, Mao L, Quail P, Wing R, Dean R, Yu Y, Zharkikh A, Shen R, Sahasrabudhe S, Thomas A, Cannings R, Gutin A, Pruss D, Reid J, Tavtigian S, Mitchell J, Eldredge G, Scholl T, Miller RM, Bhatnagar S, Adey N, Rubano T, Tusneem N, Robinson R, Feldhaus J, Macalma T, Oliphant A, Briggs S (2002). A draft sequence of the rice genome (Oryza sativa L. ssp. japonica). Science.

[B25] Yu J, Hu S, Wang J, Wong GK, Li S, Liu B, Deng Y, Dai L, Zhou Y, Zhang X, Cao M, Liu J, Sun J, Tang J, Chen Y, Huang X, Lin W, Ye C, Tong W, Cong L, Geng J, Han Y, Li L, Li W, Hu G, Huang X, Li W, Li J, Liu Z, Li L, Liu J, Qi Q, Liu J, Li L, Li T, Wang X, Lu H, Wu T, Zhu M, Ni P, Han H, Dong W, Ren X, Feng X, Cui P, Li X, Wang H, Xu X, Zhai W, Xu Z, Zhang J, He S, Zhang J, Xu J, Zhang K, Zheng X, Dong J, Zeng W, Tao L, Ye J, Tan J, Ren X, Chen X, He J, Liu D, Tian W, Tian C, Xia H, Bao Q, Li G, Gao H, Cao T, Wang J, Zhao W, Li P, Chen W, Wang X, Zhang Y, Hu J, Wang J, Liu S, Yang J, Zhang G, Xiong Y, Li Z, Mao L, Zhou C, Zhu Z, Chen R, Hao B, Zheng W, Chen S, Guo W, Li G, Liu S, Tao M, Wang J, Zhu L, Yuan L, Yang H (2002). A draft sequence of the rice genome (Oryza sativa L. ssp. indica). Science.

[B26] Areshchenkova T, Ganal MW (1999). Long tomato microsatellites are predominantly associated with centromeric regions. Genome.

[B27] Van der Hoeven R, Ronning C, Giovannoni J, Martin G, Tanksley S (2002). Deductions about the number, organization, and evolution of genes in the tomato genome based on analysis of a large expressed sequence tag collection and selective genomic sequencing. The Plant Cell.

[B28] TAIR. http://www.arabidopsis.org/.

[B29] Schuler MA, Werck-Reichhart D (2003). Functional genomics of P450s. Annu Rev Plant Biol.

[B30] Nelson DR, Schuler MA, Paquette SM, Werck-Reichhart D, Bak S (2004). Comparative genomics of rice and Arabidopsis. Analysis of 727 cytochrome P450 genes and pseudogenes from a monocot and a dicot. Plant Physiol.

[B31] Xu Y, Ishida H, Reisen D, Hanson MR (2006). Upregulation of a tonoplast-localized cytochrome P450 during petal senescence in Petunia inflata. BMC Plant Biol.

[B32] Bowers JE, Chapman BA, Rong J, Paterson AH (2003). Unravelling angiosperm genome evolution by phylogenetic analysis of chromosomal duplication events. Nature.

[B33] Rossberg M, Theres K, Acarkan A, Herrero R, Schmitt T, Schumacher K, Schmitz G, Schmidt R (2001). Comparative sequence analysis reveals extensive microcolinearity in the lateral suppressor regions of the tomato, Arabidopsis, and Capsella genomes. The Plant Cell.

[B34] Ku HM, Vision T, Liu J, Tanksley SD (2000). Comparing sequenced segments of the tomato and Arabidopsis genomes: large-scale duplication followed by selective gene loss creates a network of synteny. Proc Natl Acad Sci U S A.

[B35] Ballvora A, Jöcker A, Viehöver P, Ishihara H, Paal J, Meksem K, Bruggmann R, Schoof H, Weisshaar B, Gebhardt C (2007). Comparative sequence analysis of Solanum and Arabidopsis in a hot spot for pathogen resistance on potato chromosome V reveals a patchwork of conserved and rapidly evolving genome segments. BMC Genomics.

[B36] Gebhardt C, Walkemeier B, Henselewski H, Barakat A, Delseny M, Stüber K (2003). Comparative mapping between potato (Solanum tuberosum) and Arabidopsis thaliana reveals structurally conserved domains and ancient duplications in the potato genome. The Plant Journal.

[B37] SOL Genomics Network. ftp://ftp.sgn.cornell.edu/.

[B38] TAIR. ftp://ftp.arabidopsis.org/home/tair/Sequences/.

[B39] NCBI dbGSS. http://www.ncbi.nlm.nih.gov/dbGSS/.

[B40] Altschul SF, Madden TL, Schaffer AA, Zhang J, Zhang Z, Miller W, Lipman DJ (1997). Gapped BLAST and PSI-BLAST: a new generation of protein database search programs. Nucleic Acids Res.

[B41] Chung HJ, Jung DJ, Park HW, Kim JH, Cha HW, Min SR, Jeong WJ, Liu JR (2006). The complete chloroplast genome sequences of Solanum tuberosum and comparative analysis with Solanaceae species identified the presence of a 241-bp deletion in cultivated potato chloroplast DNA sequence. Plant Cell Reports.

[B42] Daniell H, Lee SB, Grevich J, Saski C, Quesada-Vargas T, Guda C, Tomkins J, Jansen RK (2006). Complete chloroplast genome sequences of Solanum bulbocastanum, Solanum lycopersicum and comparative analyses with other Solanaceae genomes. Theoretical and Applied Genetics.

[B43] Jurka J, Kapitonov VV, Pavlicek A, Klonowski P, Kohany O, Walichiewicz J (2005). Repbase Update, a database of eukaryotic repetitive elements. Cytogenet Genome Res.

[B44] RepeatMasker. http://www.repeatmasker.org/.

[B45] Green Group. http://www.phrap.org/.

[B46] EST-SSRs From Wheat, Barley And Rice. http://wheat.pw.usda.gov/ITMI/EST-SSR/LaRota/.

[B47] GenBank. http://www.ncbi.nlm.nih.gov/Genbank/.

[B48] Micro-Tom Database. http://www.kazusa.or.jp/jsol/microtom/.

[B49] Finn RD, Mistry J, Schuster-Bockler B, Griffiths-Jones S, Hollich V, Lassmann T, Moxon S, Marshall M, Khanna A, Durbin R, Eddy SR, Sonnhammer EL, Bateman A (2006). Pfam: clans, web tools and services. Nucleic Acids Res.

[B50] Mi H, Guo N, Kejariwal A, Thomas PD (2007). PANTHER version 6: protein sequence and function evolution data with expanded representation of biological pathways. Nucleic Acids Res.

[B51] Mulder NJ, Apweiler R, Attwood TK, Bairoch A, Bateman A, Binns D, Bork P, Buillard V, Cerutti L, Copley R, Courcelle E, Das U, Daugherty L, Dibley M, Finn R, Fleischmann W, Gough J, Haft D, Hulo N, Hunter S, Kahn D, Kanapin A, Kejariwal A, Labarga A, Langendijk-Genevaux PS, Lonsdale D, Lopez R, Letunic I, Madera M, Maslen J, McAnulla C, McDowall J, Mistry J, Mitchell A, Nikolskaya AN, Orchard S, Orengo C, Petryszak R, Selengut JD, Sigrist CJ, Thomas PD, Valentin F, Wilson D, Wu CH, Yeats C (2007). New developments in the InterPro database. Nucleic Acids Res.

[B52] The R Project For Statistical Computing. http://www.r-project.org/.

[B53] Joint Genome Institute. ftp://ftp.jgi-psf.org/pub/JGI_data/Poplar/.

