# Supplementary material for: Comparative BAC end sequence analysis of tomato and potato reveals overrepresentation of specific gene families in potato
Source: BMC Plant Biol. 2008 Apr 11;8:34. doi: 10.1186/1471-2229-8-34 (PMC2324086; doi:10.1186/1471-2229-8-34)
Supplement: Additional file 1 — This file describes the keyword filtering that has been applied after the BLASTX searches to the non-redundant protein database, in order to distinguish between true putative protein-coding regions, and repetitive and/or contamination-related sequences. [file 1471-2229-8-34-S1.doc]

**Blastx keyword filtering**

In order to distinguish between true putative protein-coding regions, and repetitive and/or contamination-related sequences, the results of the blastx comparison to the non-redundant protein database have been filtered, based on keyword terms in the description field of the database sequences. The following pseudo-code briefly describes the keyword filtering:

For each BAC end sequence

description_words = getUniqueWordsFromBlastxHitsDescriptions()

If contamination_terms in description_words:

Category = “contamination”

Else if chloroplast_terms in description_words:

Category = “chloroplast”

Else if mitochondrial_terms in description_words:

Category = “mitochondrial”

Else if ribosomal_terms in description_words:

Category = “ribosomal”

Else if transposon_terms in description_words:

Category = “transposon”

Else:

Category = “putative_genes”

Thus, a BAC end sequence is only assigned to the “putative_genes” category, if none of the blastx hits to that sequence have terms in their description fields (description_words) that match any of the keyword terms (one of contamination_terms, chloroplast_terms, mitochondrial_terms, ribosomal_terms, transposon_terms). In order for a keyword term to match a blastx description, all words in that keyword term must be present in the description of the blastx hit. Here, we do not take into account the case and order of the words in the keyword term, compared to those of the blastx description.

For a list of keyword terms corresponding to each of the categories, see below. Please note that these lists have been created through manual inspection of the blastx results and iterative refinement; however, they are not necessarily exhaustive. Also note that the list of ribosomal terms includes two accession numbers; these sequences do not have any description associated to them in the protein database but are highly similar to an endonuclease (xp_001005117.1) and a transcribed rRNA spaces sequence (bae98425.1), and produce a large number of hits on the BAC end sequences.

**Keyword terms**

Contamination terms

- Escherichia coli
- E coli
- Plasmid
- Cloning vector
- Replication initiation

Chloroplast terms

- Chloroplast
- Plastid

Mitochondrial terms

- Mitochondrium
- Mitochondria
- Mitochondrial

Ribosomal terms

- Ribosomal RNA
- Ribosomal DNA
- rRNA
- rDNA
- rRNA intron encoded homing endonuclease
- xp_001005117.1
- bae98425.1

Transposon terms

- Transposon
- Retrotransposon
- Transposable element
- Retrotransposable element
- Retroelement
- Polyprotein
- Gag
- Pol
- Copia
- Gypsy
- Integrase
- Reverse transcriptase
- RNA directed DNA polymerase
- RNase H
- Ribonuclease H
- Translocation
- Transactivator
